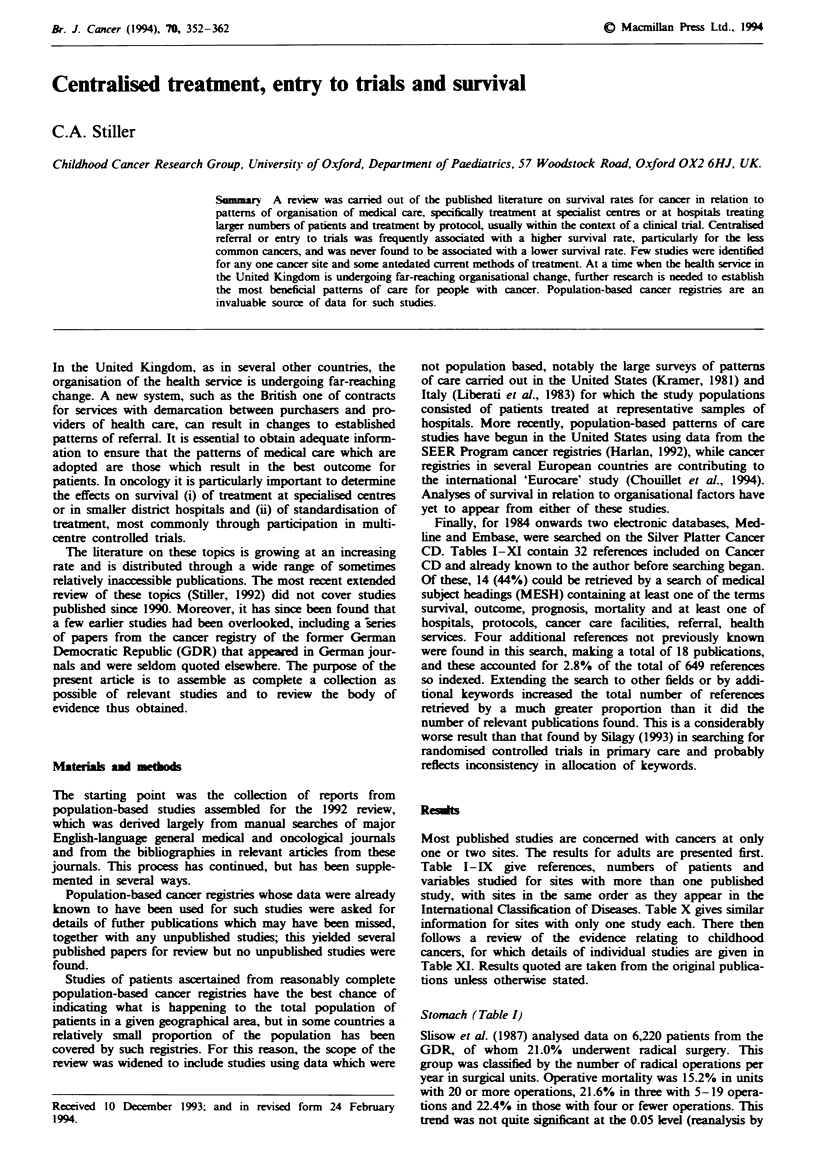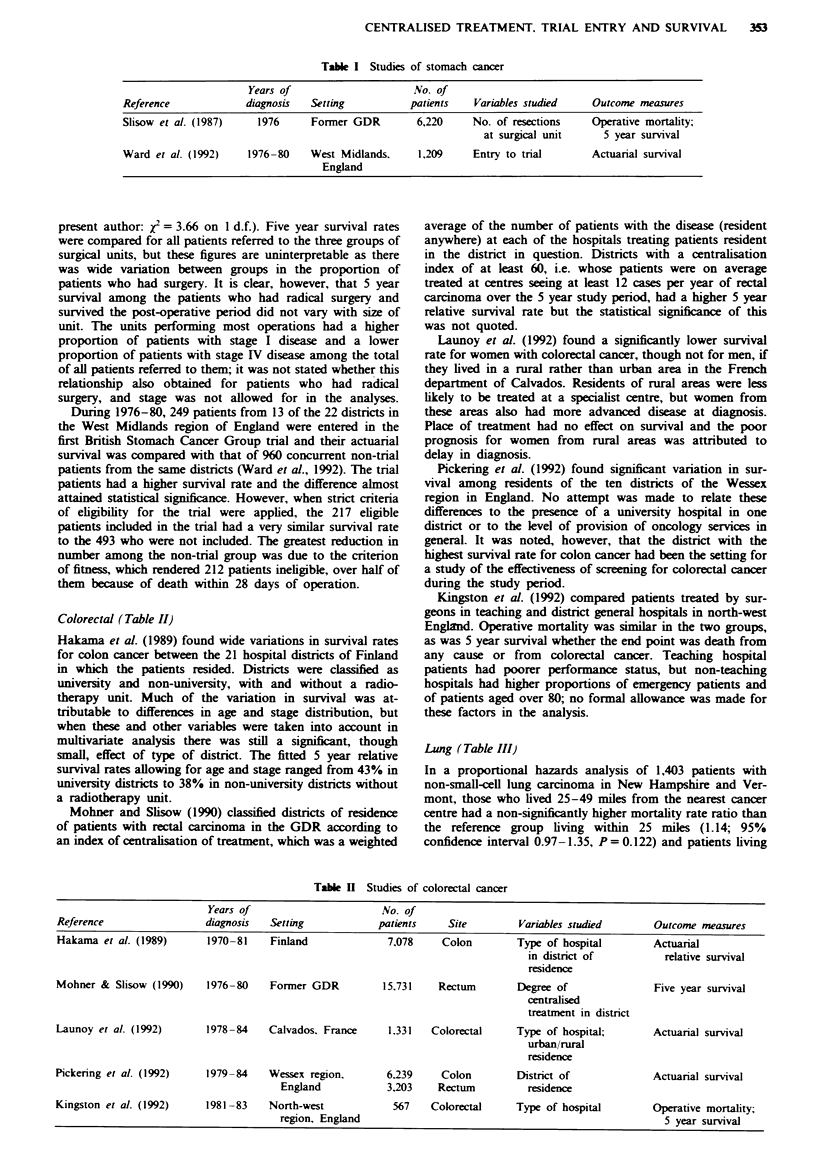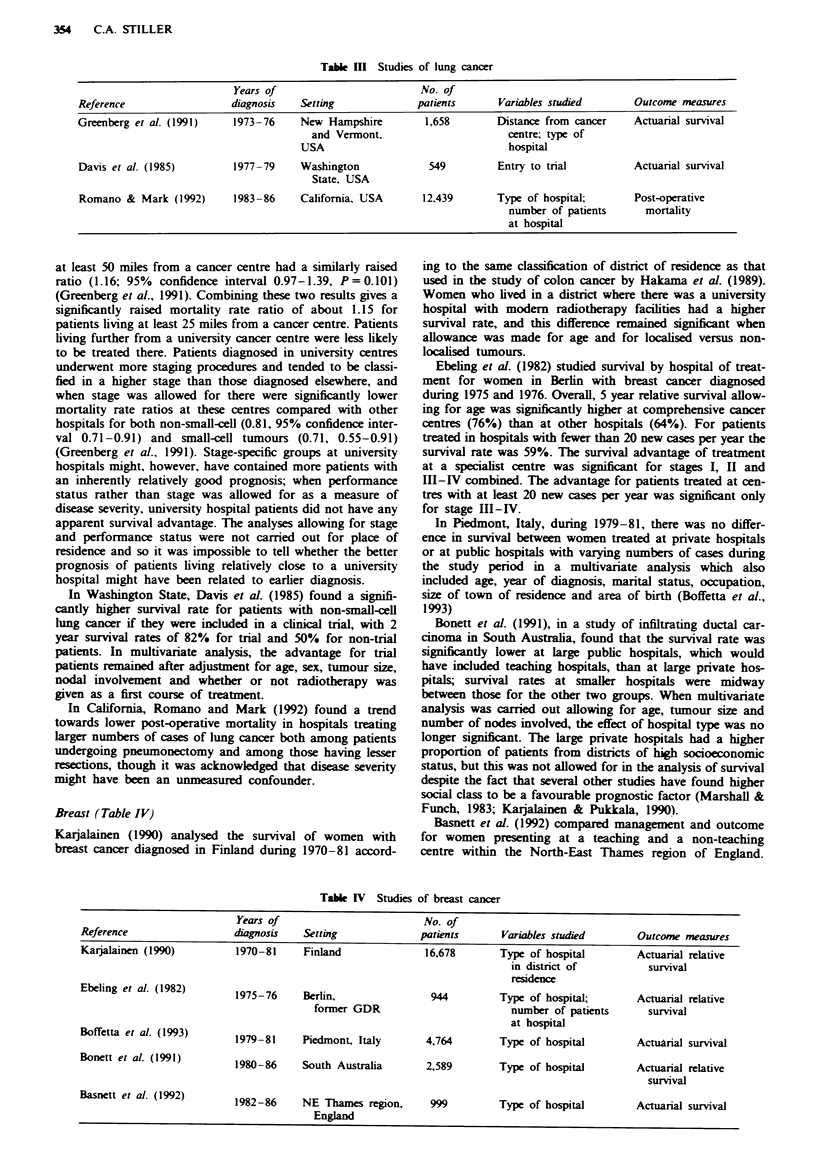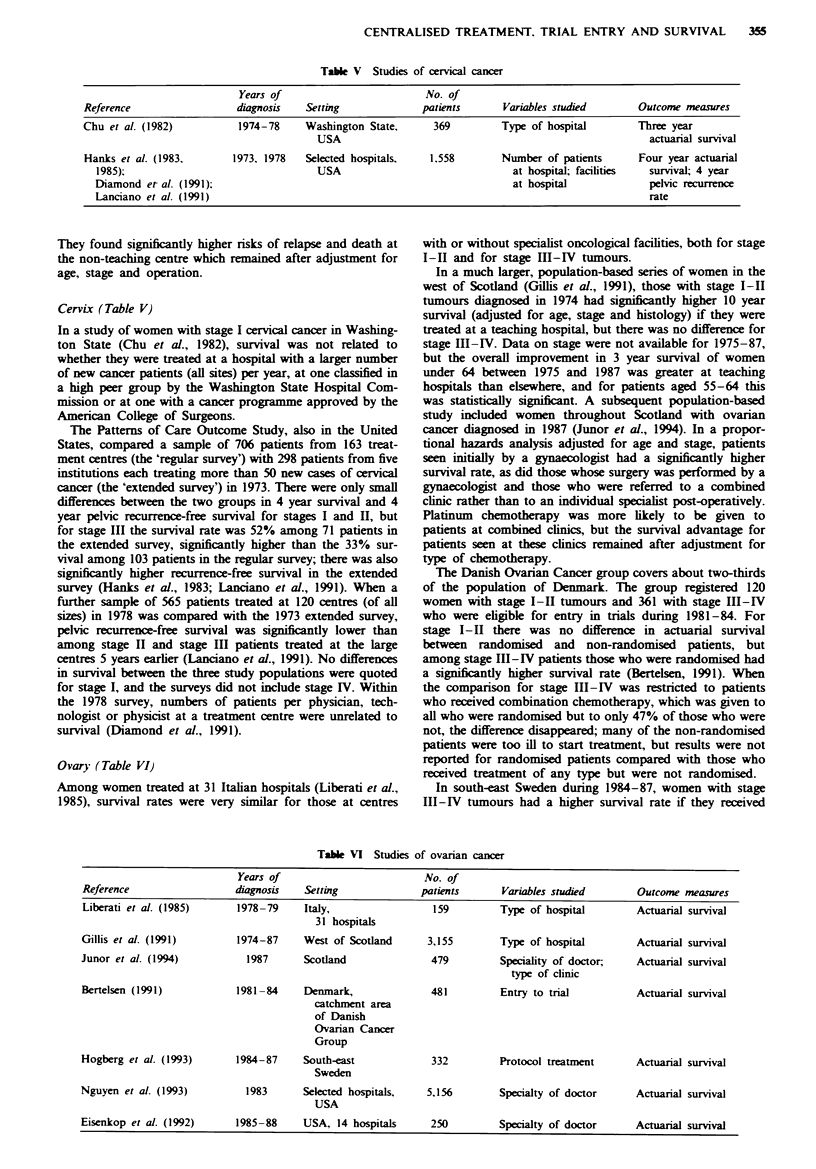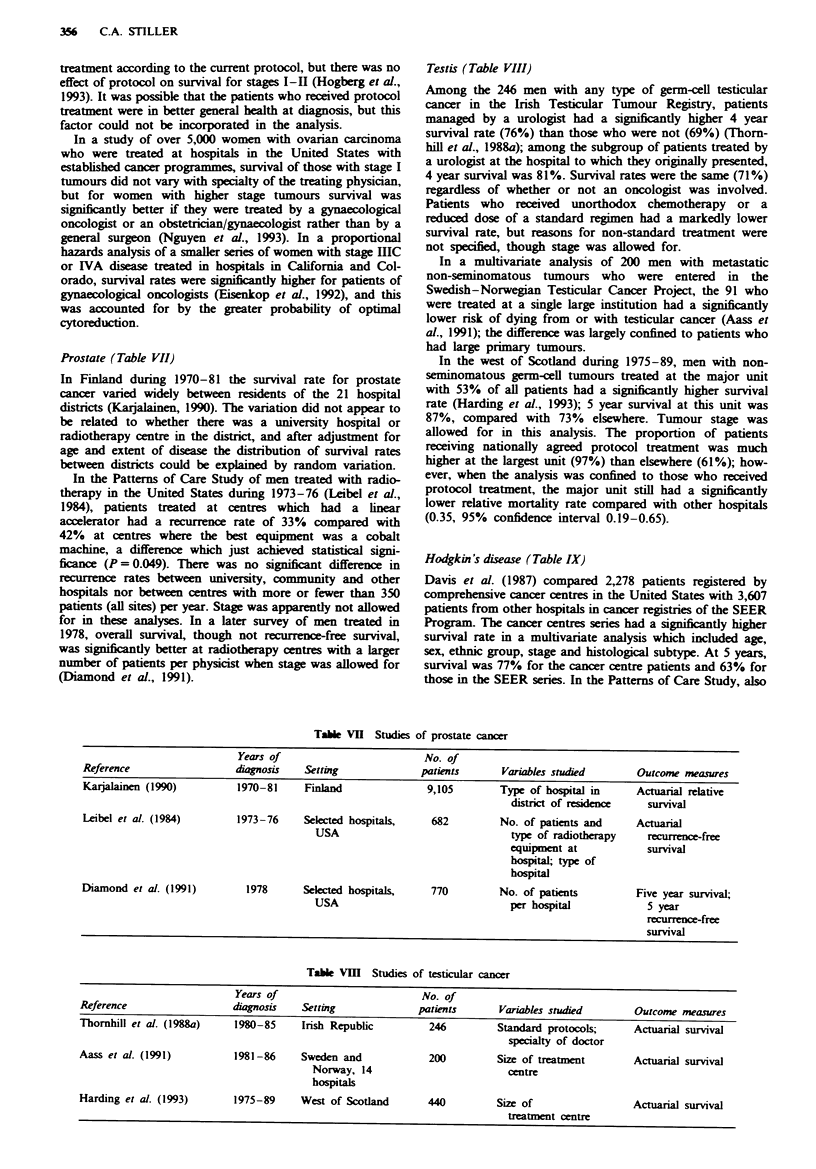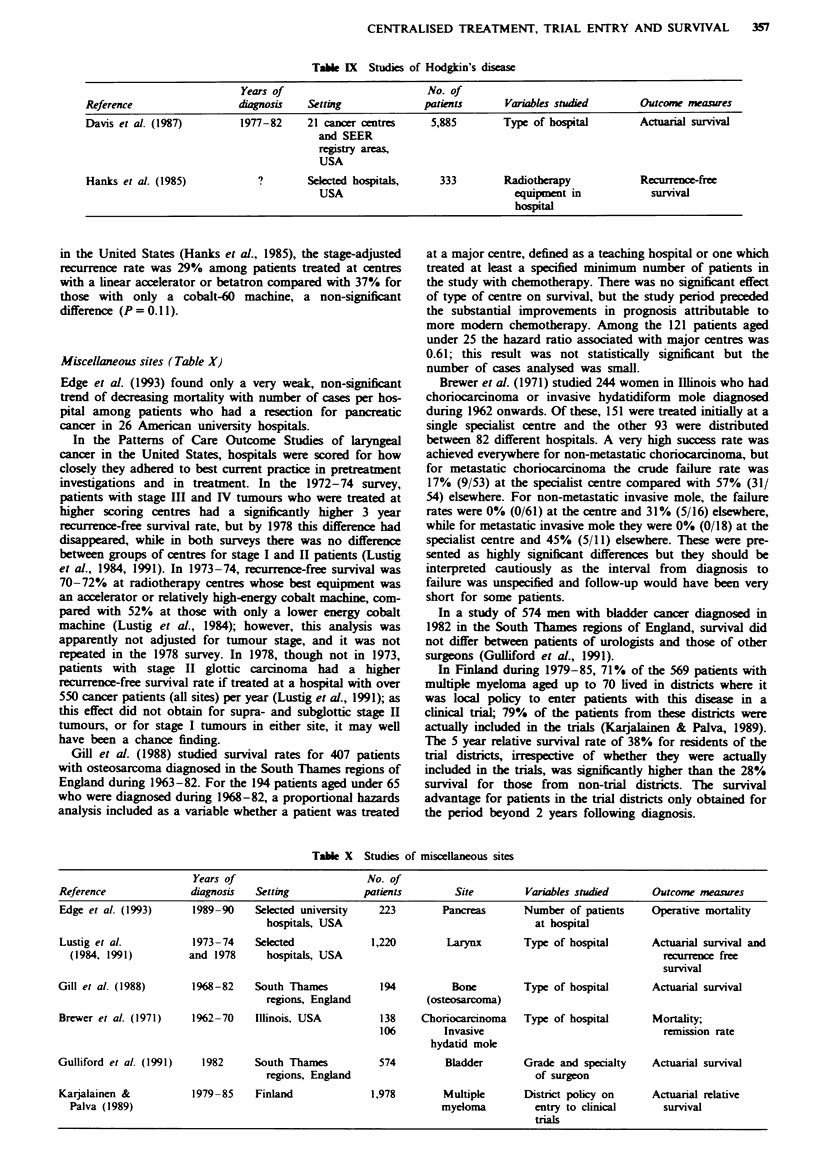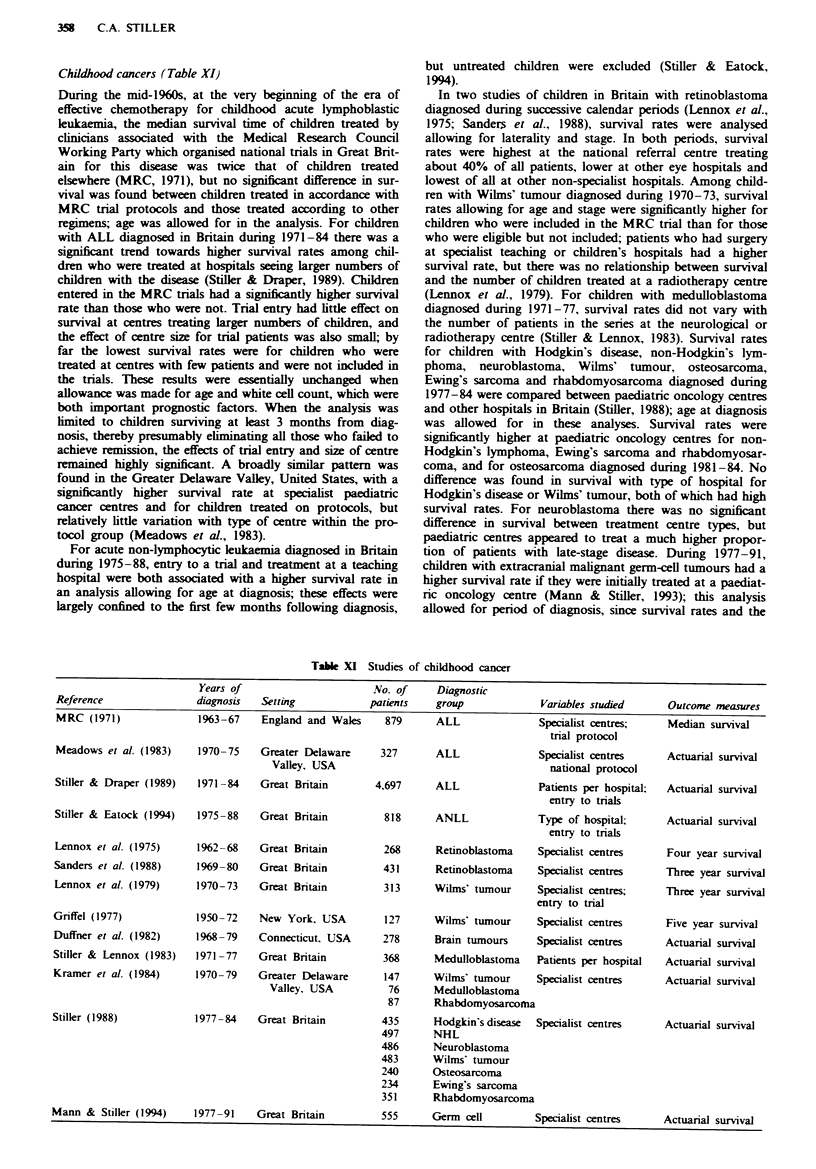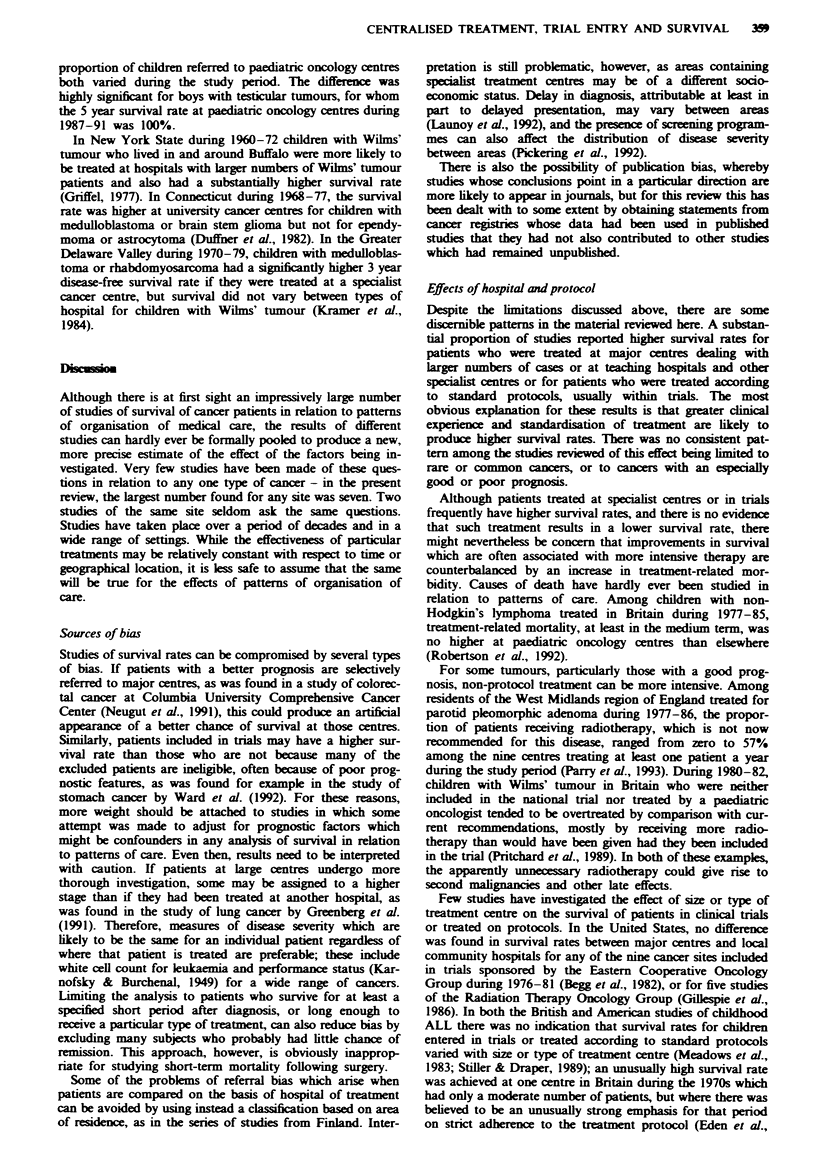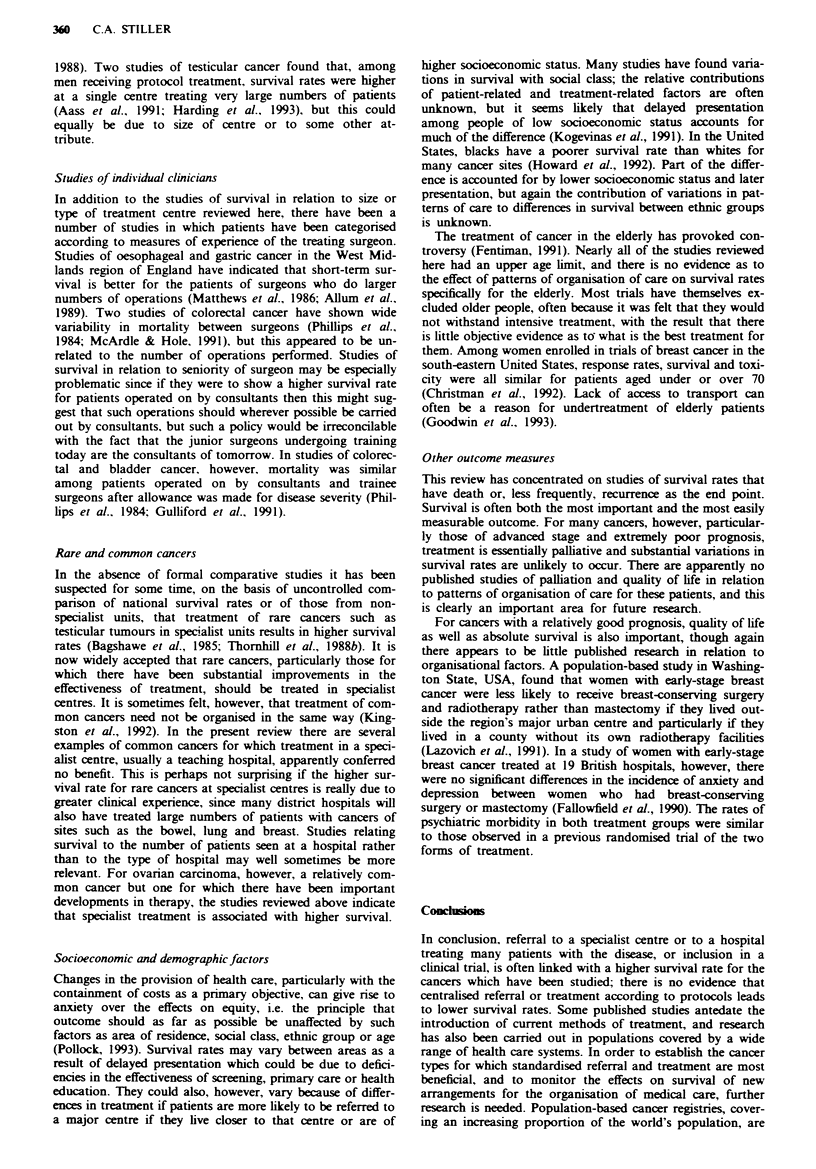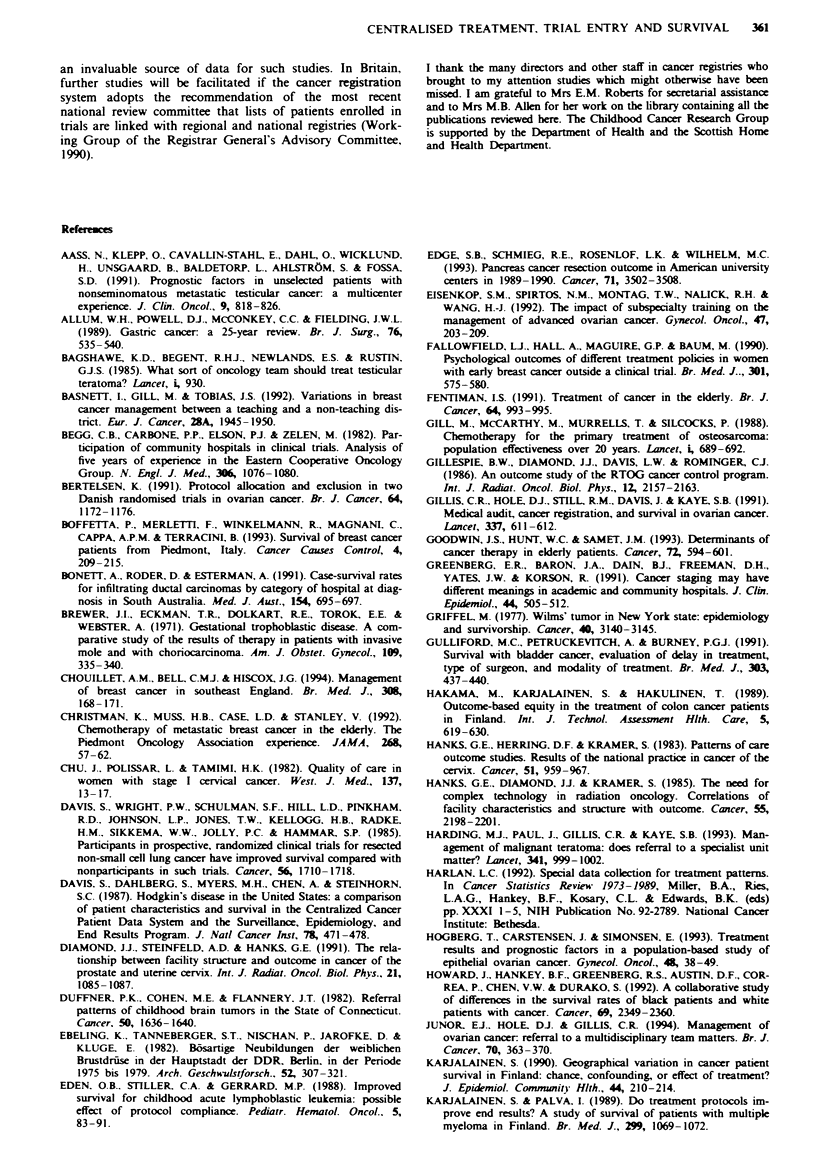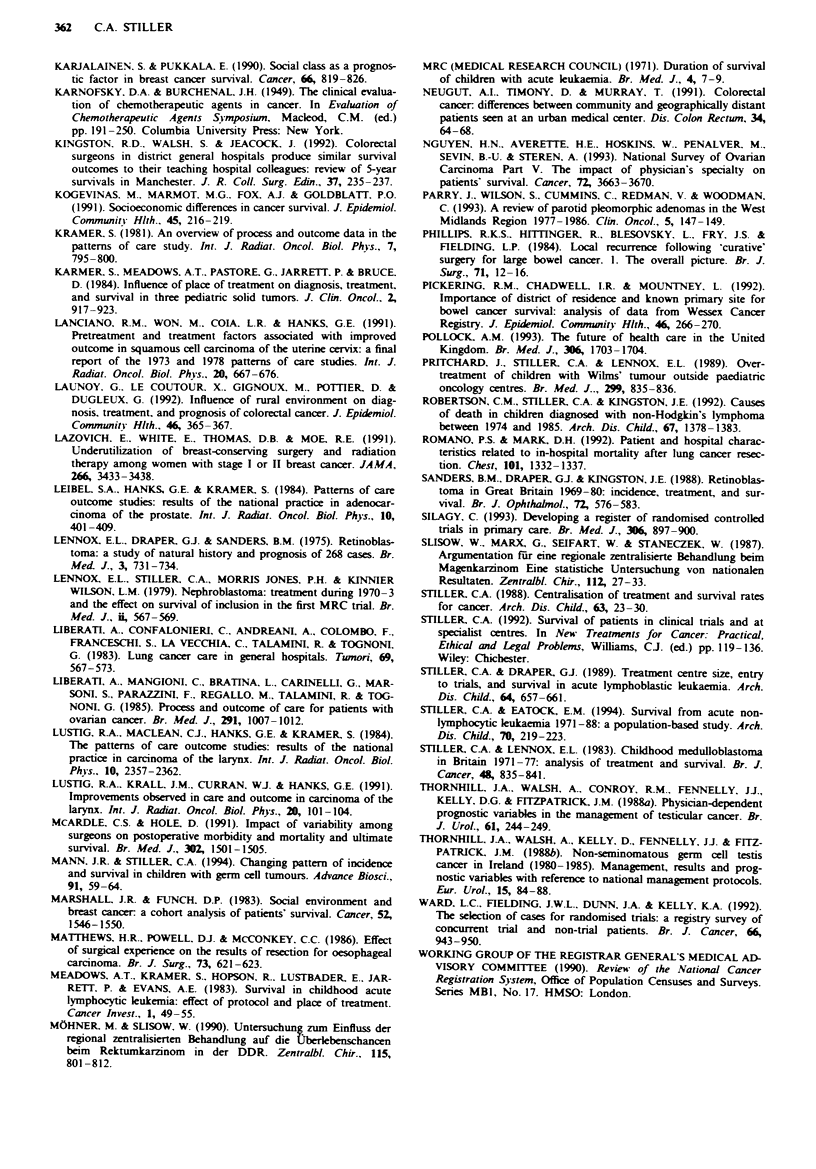# Centralised treatment, entry to trials and survival.

**DOI:** 10.1038/bjc.1994.306

**Published:** 1994-08

**Authors:** C. A. Stiller

**Affiliations:** University of Oxford, Department of Paediatrics, UK.

## Abstract

A review was carried out of the published literature on survival rates for cancer in relation to patterns of organisation of medical care, specifically treatment at specialist centres or at hospitals treating larger numbers of patients and treatment by protocol, usually within the context of a clinical trial. Centralised referral or entry to trials was frequently associated with a higher survival rate, particularly for the less common cancers, and was never found to be associated with a lower survival rate. Few studies were identified for any one cancer site and some antedated current methods of treatment. At a time when the health service in the United Kingdom is undergoing far-reaching organisational change, further research is needed to establish the most beneficial patterns of care for people with cancer. Population-based cancer registries are an invaluable source of data for such studies.


Br. J. Cancer (1994), 70, 352-362 © Macmillan Press Ltd., 1994

### Centralised treatment, entry to trials and survival

C.A. Stiller

Childhood Cancer Research Group, University of Oxford, Department of Paediatrics, 57 Woodstock Road, Oxford OX2 6HJ, UK.

**Summary** A review was carried out of the published literature on survival rates for cancer in relation to patterns of organisation of medical care, specifically treatment at specialist centres or at hospitals treating larger numbers of patients and treatment by protocol, usually within the context of a clinical trial. Centralised referral or entry to trials was frequently associated with a higher survival rate, particularly for the less common cancers, and was never found to be associated with a lower survival rate. Few studies were identified for any one cancer site and some antedated current methods of treatment. At a time when the health service in the United Kingdom is undergoing far-reaching organisational change, further research is needed to establish the most beneficial patterns of care for people with cancer. Population-based cancer registries are an invaluable source of data for such studies.

In the United Kingdom, as in several other countries, the organisation of the health service is undergoing far-reaching change. A new system, such as the British one of contracts for services with demarcation between purchasers and providers of health care, can result in changes to established patterns of referral. It is essential to obtain adequate information to ensure that the patterns of medical care which are adopted are those which result in the best outcome for patients. In oncology it is particularly important to determine the effects on survival (i) of treatment at specialised centres or in smaller district hospitals and (ii) of standardisation of treatment, most commonly through participation in multicentre controlled trials.

The literature on these topics is growing at an increasing rate and is distributed through a wide range of sometimes relatively inaccessible publications. The most recent extended review of these topics (Stiller, 1992) did not cover studies published since 1990. Moreover, it has since been found that a few earlier studies had been overlooked, including a series of papers from the cancer registry of the former German Democratic Republic (GDR) that appeared in German journals and were seldom quoted elsewhere. The purpose of the present article is to assemble as complete a collection as possible of relevant studies and to review the body of evidence thus obtained.

### Materials and methods

The starting point was the collection of reports from population-based studies assembled for the 1992 review, which was derived largely from manual searches of major English-language general medical and oncological journals and from the bibliographies in relevant articles from these journals. This process has continued, but has been supplemented in several ways.

Population-based cancer registries whose data were already known to have been used for such studies were asked for details of futher publications which may have been missed, together with any unpublished studies; this yielded several published papers for review but no unpublished studies were found.

Studies of patients ascertained from reasonably complete population-based cancer registries have the best chance of indicating what is happening to the total population of patients in a given geographical area, but in some countries a relatively small proportion of the population has been covered by such registries. For this reason, the scope of the review was widened to include studies using data which were not population based, notably the large surveys of patterns of care carried out in the United States (Kramer, 1981) and Italy (Liberati *et al.*, 1983) for which the study populations consisted of patients treated at representative samples of hospitals. More recently, population-based patterns of care studies have begun in the United States using data from the SEER Program cancer registries (Harlan, 1992), while cancer registries in several European countries are contributing to the international 'Eurocare' study (Chouillet *et al.*, 1994). Analyses of survival in relation to organisational factors have yet to appear from either of these studies.

Finally, for 1984 onwards two electronic databases, Medline and Embase, were searched on the Silver Platter Cancer CD. Tables I-XI contain 32 references included on Cancer CD and already known to the author before searching began. Of these, 14 (44%) could be retrieved by a search of medical subject headings (MESH) containing at least one of the terms survival, outcome, prognosis, mortality and at least one of hospitals, protocols, cancer care facilities, referral, health services. Four additional references not previously known were found in this search, making a total of 18 publications, and these accounted for 2.8% of the total of 649 references so indexed. Extending the search to other fields or by additional keywords increased the total number of references retrieved by a much greater proportion than it did the number of relevant publications found. This is a considerably worse result than that found by Silagy (1993) in searching for randomised controlled trials in primary care and probably reflects inconsistency in allocation of keywords.

### Results

Most published studies are concerned with cancers at only one or two sites. The results for adults are presented first. Table I-IX give references, numbers of patients and variables studied for sites with more than one published study, with sites in the same order as they appear in the International Classification of Diseases. Table X gives similar information for sites with only one study each. There then follows a review of the evidence relating to childhood cancers, for which details of individual studies are given in Table XI. Results quoted are taken from the original publications unless otherwise stated.

### *Stomach (Table I)*

Slisow *et al.* (1987) analysed data on 6,220 patients from the GDR, of whom 21.0% underwent radical surgery. This group was classified by the number of radical operations per year in surgical units. Operative mortality was 15.2% in units with 20 or more operations, 21.6% in three with 5-19 operations and 22.4% in those with four or fewer operations. This trend was not quite significant at the 0.05 level (reanalysis by

Received 10 December 1993; and in revised form 24 February 1994.

**Table I** Studies of stomach cancer

| *Reference* | *Years of diagnosis* | *Setting* | *No. of patients* | *Variables studied* | *Outcome measures* |
|---|---|---|---|---|---|
| Slisow *et al.* (1987) | 1976 | Former GDR | 6,220 | No. of resections at surgical unit | Operative mortality; 5 year survival |
| Ward *et al.* (1992) | 1976–80 | West Midlands, England | 1,209 | Entry to trial | Actuarial survival |

present author: $\chi^2 = 3.66$ on 1 d.f.). Five year survival rates were compared for all patients referred to the three groups of surgical units, but these figures are uninterpretable as there was wide variation between groups in the proportion of patients who had surgery. It is clear, however, that 5 year survival among the patients who had radical surgery and survived the post-operative period did not vary with size of unit. The units performing most operations had a higher proportion of patients with stage I disease and a lower proportion of patients with stage IV disease among the total of all patients referred to them; it was not stated whether this relationship also obtained for patients who had radical surgery, and stage was not allowed for in the analyses.

During 1976–80, 249 patients from 13 of the 22 districts in the West Midlands region of England were entered in the first British Stomach Cancer Group trial and their actuarial survival was compared with that of 960 concurrent non-trial patients from the same districts (Ward *et al.*, 1992). The trial patients had a higher survival rate and the difference almost attained statistical significance. However, when strict criteria of eligibility for the trial were applied, the 217 eligible patients included in the trial had a very similar survival rate to the 493 who were not included. The greatest reduction in number among the non-trial group was due to the criterion of fitness, which rendered 212 patients ineligible, over half of them because of death within 28 days of operation.

*Colorectal (Table II)*

Hakama *et al.* (1989) found wide variations in survival rates for colon cancer between the 21 hospital districts of Finland in which the patients resided. Districts were classified as university and non-university, with and without a radiotherapy unit. Much of the variation in survival was attributable to differences in age and stage distribution, but when these and other variables were taken into account in multivariate analysis there was still a significant, though small, effect of type of district. The fitted 5 year relative survival rates allowing for age and stage ranged from 43% in university districts to 38% in non-university districts without a radiotherapy unit.

Mohner and Slisow (1990) classified districts of residence of patients with rectal carcinoma in the GDR according to an index of centralisation of treatment, which was a weighted average of the number of patients with the disease (resident anywhere) at each of the hospitals treating patients resident in the district in question. Districts with a centralisation index of at least 60, i.e. whose patients were on average treated at centres seeing at least 12 cases per year of rectal carcinoma over the 5 year study period, had a higher 5 year relative survival rate but the statistical significance of this was not quoted.

Launoy *et al.* (1992) found a significantly lower survival rate for women with colorectal cancer, though not for men, if they lived in a rural rather than urban area in the French department of Calvados. Residents of rural areas were less likely to be treated at a specialist centre, but women from these areas also had more advanced disease at diagnosis. Place of treatment had no effect on survival and the poor prognosis for women from rural areas was attributed to delay in diagnosis.

Pickering *et al.* (1992) found significant variation in survival among residents of the ten districts of the Wessex region in England. No attempt was made to relate these differences to the presence of a university hospital in one district or to the level of provision of oncology services in general. It was noted, however, that the district with the highest survival rate for colon cancer had been the setting for a study of the effectiveness of screening for colorectal cancer during the study period.

Kingston *et al.* (1992) compared patients treated by surgeons in teaching and district general hospitals in north-west England. Operative mortality was similar in the two groups, as was 5 year survival whether the end point was death from any cause or from colorectal cancer. Teaching hospital patients had poorer performance status, but non-teaching hospitals had higher proportions of emergency patients and of patients aged over 80; no formal allowance was made for these factors in the analysis.

*Lung (Table III)*

In a proportional hazards analysis of 1,403 patients with non-small-cell lung carcinoma in New Hampshire and Vermont, those who lived 25–49 miles from the nearest cancer centre had a non-significantly higher mortality rate ratio than the reference group living within 25 miles (1.14; 95% confidence interval 0.97–1.35, $P = 0.122$) and patients living

**Table II** Studies of colorectal cancer

| *Reference* | *Years of diagnosis* | *Setting* | *No. of patients* | *Site* | *Variables studied* | *Outcome measures* |
|---|---|---|---|---|---|---|
| Hakama *et al.* (1989) | 1970–81 | Finland | 7,078 | Colon | Type of hospital in district of residence | Actuarial relative survival |
| Mohner & Slisow (1990) | 1976–80 | Former GDR | 15,731 | Rectum | Degree of centralised treatment in district | Five year survival |
| Launoy *et al.* (1992) | 1978–84 | Calvados, France | 1,331 | Colorectal | Type of hospital; urban/rural residence | Actuarial survival |
| Pickering *et al.* (1992) | 1979–84 | Wessex region, England | 6,239<br>3,203 | Colon<br>Rectum | District of residence | Actuarial survival |
| Kingston *et al.* (1992) | 1981–83 | North-west region, England | 567 | Colorectal | Type of hospital | Operative mortality; 5 year survival |

Table III Studies of lung cancer

| Reference | Years of diagnosis | Setting | No. of patients | Variables studied | Outcome measures |
|---|---|---|---|---|---|
| Greenberg *et al.* (1991) | 1973–76 | New Hampshire and Vermont, USA | 1,658 | Distance from cancer centre; type of hospital | Actuarial survival |
| Davis *et al.* (1985) | 1977–79 | Washington State, USA | 549 | Entry to trial | Actuarial survival |
| Romano & Mark (1992) | 1983–86 | California, USA | 12,439 | Type of hospital; number of patients at hospital | Post-operative mortality |

at least 50 miles from a cancer centre had a similarly raised ratio (1.16; 95% confidence interval 0.97–1.39, $P = 0.101$) (Greenberg *et al.*, 1991). Combining these two results gives a significantly raised mortality rate ratio of about 1.15 for patients living at least 25 miles from a cancer centre. Patients living further from a university cancer centre were less likely to be treated there. Patients diagnosed in university centres underwent more staging procedures and tended to be classified in a higher stage than those diagnosed elsewhere, and when stage was allowed for there were significantly lower mortality rate ratios at these centres compared with other hospitals for both non-small-cell (0.81, 95% confidence interval 0.71–0.91) and small-cell tumours (0.71, 0.55–0.91) (Greenberg *et al.*, 1991). Stage-specific groups at university hospitals might, however, have contained more patients with an inherently relatively good prognosis; when performance status rather than stage was allowed for as a measure of disease severity, university hospital patients did not have any apparent survival advantage. The analyses allowing for stage and performance status were not carried out for place of residence and so it was impossible to tell whether the better prognosis of patients living relatively close to a university hospital might have been related to earlier diagnosis.

In Washington State, Davis *et al.* (1985) found a significantly higher survival rate for patients with non-small-cell lung cancer if they were included in a clinical trial, with 2 year survival rates of 82% for trial and 50% for non-trial patients. In multivariate analysis, the advantage for trial patients remained after adjustment for age, sex, tumour size, nodal involvement and whether or not radiotherapy was given as a first course of treatment.

In California, Romano and Mark (1992) found a trend towards lower post-operative mortality in hospitals treating larger numbers of cases of lung cancer both among patients undergoing pneumonectomy and among those having lesser resections, though it was acknowledged that disease severity might have been an unmeasured confounder.

### *Breast (Table IV)*

Karjalainen (1990) analysed the survival of women with breast cancer diagnosed in Finland during 1970–81 according to the same classification of district of residence as that used in the study of colon cancer by Hakama *et al.* (1989). Women who lived in a district where there was a university hospital with modern radiotherapy facilities had a higher survival rate, and this difference remained significant when allowance was made for age and for localised versus non-localised tumours.

Ebeling *et al.* (1982) studied survival by hospital of treatment for women in Berlin with breast cancer diagnosed during 1975 and 1976. Overall, 5 year relative survival allowing for age was significantly higher at comprehensive cancer centres (76%) than at other hospitals (64%). For patients treated in hospitals with fewer than 20 new cases per year the survival rate was 59%. The survival advantage of treatment at a specialist centre was significant for stages I, II and III–IV combined. The advantage for patients treated at centres with at least 20 new cases per year was significant only for stage III–IV.

In Piedmont, Italy, during 1979–81, there was no difference in survival between women treated at private hospitals or at public hospitals with varying numbers of cases during the study period in a multivariate analysis which also included age, year of diagnosis, marital status, occupation, size of town of residence and area of birth (Boffetta *et al.*, 1993)

Bonett *et al.* (1991), in a study of infiltrating ductal carcinoma in South Australia, found that the survival rate was significantly lower at large public hospitals, which would have included teaching hospitals, than at large private hospitals; survival rates at smaller hospitals were midway between those for the other two groups. When multivariate analysis was carried out allowing for age, tumour size and number of nodes involved, the effect of hospital type was no longer significant. The large private hospitals had a higher proportion of patients from districts of high socioeconomic status, but this was not allowed for in the analysis of survival despite the fact that several other studies have found higher social class to be a favourable prognostic factor (Marshall & Funch, 1983; Karjalainen & Pukkala, 1990).

Basnett *et al.* (1992) compared management and outcome for women presenting at a teaching and a non-teaching centre within the North-East Thames region of England.

Table IV Studies of breast cancer

| Reference | Years of diagnosis | Setting | No. of patients | Variables studied | Outcome measures |
|---|---|---|---|---|---|
| Karjalainen (1990) | 1970–81 | Finland | 16,678 | Type of hospital in district of residence | Actuarial relative survival |
| Ebeling *et al.* (1982) | 1975–76 | Berlin, former GDR | 944 | Type of hospital; number of patients at hospital | Actuarial relative survival |
| Boffetta *et al.* (1993) | 1979–81 | Piedmont, Italy | 4,764 | Type of hospital | Actuarial survival |
| Bonett *et al.* (1991) | 1980–86 | South Australia | 2,589 | Type of hospital | Actuarial relative survival |
| Basnett *et al.* (1992) | 1982–86 | NE Thames region, England | 999 | Type of hospital | Actuarial survival |

**Table V** Studies of cervical cancer

| Reference | Years of diagnosis | Setting | No. of patients | Variables studied | Outcome measures |
|---|---|---|---|---|---|
| Chu *et al.* (1982) | 1974–78 | Washington State, USA | 369 | Type of hospital | Three year actuarial survival |
| Hanks *et al.* (1983, 1985); Diamond *et al.* (1991); Lanciano *et al.* (1991) | 1973, 1978 | Selected hospitals, USA | 1,558 | Number of patients at hospital; facilities at hospital | Four year actuarial survival; 4 year pelvic recurrence rate |

They found significantly higher risks of relapse and death at the non-teaching centre which remained after adjustment for age, stage and operation.

*Cervix (Table V)*

In a study of women with stage I cervical cancer in Washington State (Chu *et al.*, 1982), survival was not related to whether they were treated at a hospital with a larger number of new cancer patients (all sites) per year, at one classified in a high peer group by the Washington State Hospital Commission or at one with a cancer programme approved by the American College of Surgeons.

The Patterns of Care Outcome Study, also in the United States, compared a sample of 706 patients from 163 treatment centres (the 'regular survey') with 298 patients from five institutions each treating more than 50 new cases of cervical cancer (the 'extended survey') in 1973. There were only small differences between the two groups in 4 year survival and 4 year pelvic recurrence-free survival for stages I and II, but for stage III the survival rate was 52% among 71 patients in the extended survey, significantly higher than the 33% survival among 103 patients in the regular survey; there was also significantly higher recurrence-free survival in the extended survey (Hanks *et al.*, 1983; Lanciano *et al.*, 1991). When a further sample of 565 patients treated at 120 centres (of all sizes) in 1978 was compared with the 1973 extended survey, pelvic recurrence-free survival was significantly lower than among stage II and stage III patients treated at the large centres 5 years earlier (Lanciano *et al.*, 1991). No differences in survival between the three study populations were quoted for stage I, and the surveys did not include stage IV. Within the 1978 survey, numbers of patients per physician, technologist or physicist at a treatment centre were unrelated to survival (Diamond *et al.*, 1991).

*Ovary (Table VI)*

Among women treated at 31 Italian hospitals (Liberati *et al.*, 1985), survival rates were very similar for those at centres with or without specialist oncological facilities, both for stage I–II and for stage III–IV tumours.

In a much larger, population-based series of women in the west of Scotland (Gillis *et al.*, 1991), those with stage I–II tumours diagnosed in 1974 had significantly higher 10 year survival (adjusted for age, stage and histology) if they were treated at a teaching hospital, but there was no difference for stage III–IV. Data on stage were not available for 1975–87, but the overall improvement in 3 year survival of women under 64 between 1975 and 1987 was greater at teaching hospitals than elsewhere, and for patients aged 55–64 this was statistically significant. A subsequent population-based study included women throughout Scotland with ovarian cancer diagnosed in 1987 (Junor *et al.*, 1994). In a proportional hazards analysis adjusted for age and stage, patients seen initially by a gynaecologist had a significantly higher survival rate, as did those whose surgery was performed by a gynaecologist and those who were referred to a combined clinic rather than to an individual specialist post-operatively. Platinum chemotherapy was more likely to be given to patients at combined clinics, but the survival advantage for patients seen at these clinics remained after adjustment for type of chemotherapy.

The Danish Ovarian Cancer group covers about two-thirds of the population of Denmark. The group registered 120 women with stage I–II tumours and 361 with stage III–IV who were eligible for entry in trials during 1981–84. For stage I–II there was no difference in actuarial survival between randomised and non-randomised patients, but among stage III–IV patients those who were randomised had a significantly higher survival rate (Bertelsen, 1991). When the comparison for stage III–IV was restricted to patients who received combination chemotherapy, which was given to all who were randomised but to only 47% of those who were not, the difference disappeared; many of the non-randomised patients were too ill to start treatment, but results were not reported for randomised patients compared with those who received treatment of any type but were not randomised.

In south-east Sweden during 1984–87, women with stage III–IV tumours had a higher survival rate if they received

**Table VI** Studies of ovarian cancer

| Reference | Years of diagnosis | Setting | No. of patients | Variables studied | Outcome measures |
|---|---|---|---|---|---|
| Liberati *et al.* (1985) | 1978–79 | Italy, 31 hospitals | 159 | Type of hospital | Actuarial survival |
| Gillis *et al.* (1991) | 1974–87 | West of Scotland | 3,155 | Type of hospital | Actuarial survival |
| Junor *et al.* (1994) | 1987 | Scotland | 479 | Speciality of doctor; type of clinic | Actuarial survival |
| Bertelsen (1991) | 1981–84 | Denmark, catchment area of Danish Ovarian Cancer Group | 481 | Entry to trial | Actuarial survival |
| Hogberg *et al.* (1993) | 1984–87 | South-east Sweden | 332 | Protocol treatment | Actuarial survival |
| Nguyen *et al.* (1993) | 1983 | Selected hospitals, USA | 5,156 | Specialty of doctor | Actuarial survival |
| Eisenkop *et al.* (1992) | 1985–88 | USA, 14 hospitals | 250 | Specialty of doctor | Actuarial survival |

treatment according to the current protocol, but there was no effect of protocol on survival for stages I–II (Hogberg *et al*., 1993). It was possible that the patients who received protocol treatment were in better general health at diagnosis, but this factor could not be incorporated in the analysis.

In a study of over 5,000 women with ovarian carcinoma who were treated at hospitals in the United States with established cancer programmes, survival of those with stage I tumours did not vary with specialty of the treating physician, but for women with higher stage tumours survival was significantly better if they were treated by a gynaecological oncologist or an obstetrician/gynaecologist rather than by a general surgeon (Nguyen *et al*., 1993). In a proportional hazards analysis of a smaller series of women with stage IIIC or IVA disease treated in hospitals in California and Colorado, survival rates were significantly higher for patients of gynaecological oncologists (Eisenkop *et al*., 1992), and this was accounted for by the greater probability of optimal cytoreduction.

*Prostate (Table VII)*

In Finland during 1970–81 the survival rate for prostate cancer varied widely between residents of the 21 hospital districts (Karjalainen, 1990). The variation did not appear to be related to whether there was a university hospital or radiotherapy centre in the district, and after adjustment for age and extent of disease the distribution of survival rates between districts could be explained by random variation.

In the Patterns of Care Study of men treated with radiotherapy in the United States during 1973–76 (Leibel *et al*., 1984), patients treated at centres which had a linear accelerator had a recurrence rate of 33% compared with 42% at centres where the best equipment was a cobalt machine, a difference which just achieved statistical significance ($P = 0.049$). There was no significant difference in recurrence rates between university, community and other hospitals nor between centres with more or fewer than 350 patients (all sites) per year. Stage was apparently not allowed for in these analyses. In a later survey of men treated in 1978, overall survival, though not recurrence-free survival, was significantly better at radiotherapy centres with a larger number of patients per physicist when stage was allowed for (Diamond *et al*., 1991).

*Testis (Table VIII)*

Among the 246 men with any type of germ-cell testicular cancer in the Irish Testicular Tumour Registry, patients managed by a urologist had a significantly higher 4 year survival rate (76%) than those who were not (69%) (Thornhill *et al*., 1988*a*); among the subgroup of patients treated by a urologist at the hospital to which they originally presented, 4 year survival was 81%. Survival rates were the same (71%) regardless of whether or not an oncologist was involved. Patients who received unorthodox chemotherapy or a reduced dose of a standard regimen had a markedly lower survival rate, but reasons for non-standard treatment were not specified, though stage was allowed for.

In a multivariate analysis of 200 men with metastatic non-seminomatous tumours who were entered in the Swedish–Norwegian Testicular Cancer Project, the 91 who were treated at a single large institution had a significantly lower risk of dying from or with testicular cancer (Aass *et al*., 1991); the difference was largely confined to patients who had large primary tumours.

In the west of Scotland during 1975–89, men with non-seminomatous germ-cell tumours treated at the major unit with 53% of all patients had a significantly higher survival rate (Harding *et al*., 1993); 5 year survival at this unit was 87%, compared with 73% elsewhere. Tumour stage was allowed for in this analysis. The proportion of patients receiving nationally agreed protocol treatment was much higher at the largest unit (97%) than elsewhere (61%); however, when the analysis was confined to those who received protocol treatment, the major unit still had a significantly lower relative mortality rate compared with other hospitals (0.35, 95% confidence interval 0.19–0.65).

*Hodgkin's disease (Table IX)*

Davis *et al*. (1987) compared 2,278 patients registered by comprehensive cancer centres in the United States with 3,607 patients from other hospitals in cancer registries of the SEER Program. The cancer centres series had a significantly higher survival rate in a multivariate analysis which included age, sex, ethnic group, stage and histological subtype. At 5 years, survival was 77% for the cancer centre patients and 63% for those in the SEER series. In the Patterns of Care Study, also

**Table VII** Studies of prostate cancer

| *Reference* | *Years of diagnosis* | *Setting* | *No. of patients* | *Variables studied* | *Outcome measures* |
|---|---|---|---|---|---|
| Karjalainen (1990) | 1970–81 | Finland | 9,105 | Type of hospital in district of residence | Actuarial relative survival |
| Leibel *et al*. (1984) | 1973–76 | Selected hospitals, USA | 682 | No. of patients and type of radiotherapy equipment at hospital; type of hospital | Actuarial recurrence-free survival |
| Diamond *et al*. (1991) | 1978 | Selected hospitals, USA | 770 | No. of patients per hospital | Five year survival; 5 year recurrence-free survival |

**Table VIII** Studies of testicular cancer

| *Reference* | *Years of diagnosis* | *Setting* | *No. of patients* | *Variables studied* | *Outcome measures* |
|---|---|---|---|---|---|
| Thornhill *et al*. (1988*a*) | 1980–85 | Irish Republic | 246 | Standard protocols; specialty of doctor | Actuarial survival |
| Aass *et al*. (1991) | 1981–86 | Sweden and Norway, 14 hospitals | 200 | Size of treatment centre | Actuarial survival |
| Harding *et al*. (1993) | 1975–89 | West of Scotland | 440 | Size of treatment centre | Actuarial survival |

**Table IX** Studies of Hodgkin's disease

| *Reference* | *Years of diagnosis* | *Setting* | *No. of patients* | *Variables studied* | *Outcome measures* |
|---|---|---|---|---|---|
| Davis *et al.* (1987) | 1977-82 | 21 cancer centres and SEER registry areas, USA | 5,885 | Type of hospital | Actuarial survival |
| Hanks *et al.* (1985) | ? | Selected hospitals, USA | 333 | Radiotherapy equipment in hospital | Recurrence-free survival |

in the United States (Hanks *et al.*, 1985), the stage-adjusted recurrence rate was 29% among patients treated at centres with a linear accelerator or betatron compared with 37% for those with only a cobalt-60 machine, a non-significant difference ($P = 0.11$).

*Miscellaneous sites (Table X)*

Edge *et al.* (1993) found only a very weak, non-significant trend of decreasing mortality with number of cases per hospital among patients who had a resection for pancreatic cancer in 26 American university hospitals.

In the Patterns of Care Outcome Studies of laryngeal cancer in the United States, hospitals were scored for how closely they adhered to best current practice in pretreatment investigations and in treatment. In the 1972-74 survey, patients with stage III and IV tumours who were treated at higher scoring centres had a significantly higher 3 year recurrence-free survival rate, but by 1978 this difference had disappeared, while in both surveys there was no difference between groups of centres for stage I and II patients (Lustig *et al.*, 1984, 1991). In 1973-74, recurrence-free survival was 70-72% at radiotherapy centres whose best equipment was an accelerator or relatively high-energy cobalt machine, compared with 52% at those with only a lower energy cobalt machine (Lustig *et al.*, 1984); however, this analysis was apparently not adjusted for tumour stage, and it was not repeated in the 1978 survey. In 1978, though not in 1973, patients with stage II glottic carcinoma had a higher recurrence-free survival rate if treated at a hospital with over 550 cancer patients (all sites) per year (Lustig *et al.*, 1991); as this effect did not obtain for supra- and subglottic stage II tumours, or for stage I tumours in either site, it may well have been a chance finding.

Gill *et al.* (1988) studied survival rates for 407 patients with osteosarcoma diagnosed in the South Thames regions of England during 1963-82. For the 194 patients aged under 65 who were diagnosed during 1968-82, a proportional hazards analysis included as a variable whether a patient was treated at a major centre, defined as a teaching hospital or one which treated at least a specified minimum number of patients in the study with chemotherapy. There was no significant effect of type of centre on survival, but the study period preceded the substantial improvements in prognosis attributable to more modern chemotherapy. Among the 121 patients aged under 25 the hazard ratio associated with major centres was 0.61; this result was not statistically significant but the number of cases analysed was small.

Brewer *et al.* (1971) studied 244 women in Illinois who had choriocarcinoma or invasive hydatidiform mole diagnosed during 1962 onwards. Of these, 151 were treated initially at a single specialist centre and the other 93 were distributed between 82 different hospitals. A very high success rate was achieved everywhere for non-metastatic choriocarcinoma, but for metastatic choriocarcinoma the crude failure rate was 17% (9/53) at the specialist centre compared with 57% (31/54) elsewhere. For non-metastatic invasive mole, the failure rates were 0% (0/61) at the centre and 31% (5/16) elsewhere, while for metastatic invasive mole they were 0% (0/18) at the specialist centre and 45% (5/11) elsewhere. These were presented as highly significant differences but they should be interpreted cautiously as the interval from diagnosis to failure was unspecified and follow-up would have been very short for some patients.

In a study of 574 men with bladder cancer diagnosed in 1982 in the South Thames regions of England, survival did not differ between patients of urologists and those of other surgeons (Gulliford *et al.*, 1991).

In Finland during 1979-85, 71% of the 569 patients with multiple myeloma aged up to 70 lived in districts where it was local policy to enter patients with this disease in a clinical trial; 79% of the patients from these districts were actually included in the trials (Karjalainen & Palva, 1989). The 5 year relative survival rate of 38% for residents of the trial districts, irrespective of whether they were actually included in the trials, was significantly higher than the 28% survival for those from non-trial districts. The survival advantage for patients in the trial districts only obtained for the period beyond 2 years following diagnosis.

**Table X** Studies of miscellaneous sites

| *Reference* | *Years of diagnosis* | *Setting* | *No. of patients* | *Site* | *Variables studied* | *Outcome measures* |
|---|---|---|---|---|---|---|
| Edge *et al.* (1993) | 1989-90 | Selected university hospitals, USA | 223 | Pancreas | Number of patients at hospital | Operative mortality |
| Lustig *et al.* (1984, 1991) | 1973-74 and 1978 | Selected hospitals, USA | 1,220 | Larynx | Type of hospital | Actuarial survival and recurrence free survival |
| Gill *et al.* (1988) | 1968-82 | South Thames regions, England | 194 | Bone (osteosarcoma) | Type of hospital | Actuarial survival |
| Brewer *et al.* (1971) | 1962-70 | Illinois, USA | 138<br>106 | Choriocarcinoma<br>Invasive hydatid mole | Type of hospital | Mortality; remission rate |
| Gulliford *et al.* (1991) | 1982 | South Thames regions, England | 574 | Bladder | Grade and specialty of surgeon | Actuarial survival |
| Karjalainen & Palva (1989) | 1979-85 | Finland | 1,978 | Multiple myeloma | District policy on entry to clinical trials | Actuarial relative survival |

*Childhood cancers (Table XI)*

During the mid-1960s, at the very beginning of the era of effective chemotherapy for childhood acute lymphoblastic leukaemia, the median survival time of children treated by clinicians associated with the Medical Research Council Working Party which organised national trials in Great Britain for this disease was twice that of children treated elsewhere (MRC, 1971), but no significant difference in survival was found between children treated in accordance with MRC trial protocols and those treated according to other regimens; age was allowed for in the analysis. For children with ALL diagnosed in Britain during 1971–84 there was a significant trend towards higher survival rates among children who were treated at hospitals seeing larger numbers of children with the disease (Stiller & Draper, 1989). Children entered in the MRC trials had a significantly higher survival rate than those who were not. Trial entry had little effect on survival at centres treating larger numbers of children, and the effect of centre size for trial patients was also small; by far the lowest survival rates were for children who were treated at centres with few patients and were not included in the trials. These results were essentially unchanged when allowance was made for age and white cell count, which were both important prognostic factors. When the analysis was limited to children surviving at least 3 months from diagnosis, thereby presumably eliminating all those who failed to achieve remission, the effects of trial entry and size of centre remained highly significant. A broadly similar pattern was found in the Greater Delaware Valley, United States, with a significantly higher survival rate at specialist paediatric cancer centres and for children treated on protocols, but relatively little variation with type of centre within the protocol group (Meadows *et al.*, 1983).

For acute non-lymphocytic leukaemia diagnosed in Britain during 1975–88, entry to a trial and treatment at a teaching hospital were both associated with a higher survival rate in an analysis allowing for age at diagnosis; these effects were largely confined to the first few months following diagnosis, but untreated children were excluded (Stiller & Eatock, 1994).

In two studies of children in Britain with retinoblastoma diagnosed during successive calendar periods (Lennox *et al.*, 1975; Sanders *et al.*, 1988), survival rates were analysed allowing for laterality and stage. In both periods, survival rates were highest at the national referral centre treating about 40% of all patients, lower at other eye hospitals and lowest of all at other non-specialist hospitals. Among children with Wilms' tumour diagnosed during 1970–73, survival rates allowing for age and stage were significantly higher for children who were included in the MRC trial than for those who were eligible but not included; patients who had surgery at specialist teaching or children's hospitals had a higher survival rate, but there was no relationship between survival and the number of children treated at a radiotherapy centre (Lennox *et al.*, 1979). For children with medulloblastoma diagnosed during 1971–77, survival rates did not vary with the number of patients in the series at the neurological or radiotherapy centre (Stiller & Lennox, 1983). Survival rates for children with Hodgkin's disease, non-Hodgkin's lymphoma, neuroblastoma, Wilms' tumour, osteosarcoma, Ewing's sarcoma and rhabdomyosarcoma diagnosed during 1977–84 were compared between paediatric oncology centres and other hospitals in Britain (Stiller, 1988); age at diagnosis was allowed for in these analyses. Survival rates were significantly higher at paediatric oncology centres for non-Hodgkin's lymphoma, Ewing's sarcoma and rhabdomyosarcoma, and for osteosarcoma diagnosed during 1981–84. No difference was found in survival with type of hospital for Hodgkin's disease or Wilms' tumour, both of which had high survival rates. For neuroblastoma there was no significant difference in survival between treatment centre types, but paediatric centres appeared to treat a much higher proportion of patients with late-stage disease. During 1977–91, children with extracranial malignant germ-cell tumours had a higher survival rate if they were initially treated at a paediatric oncology centre (Mann & Stiller, 1993); this analysis allowed for period of diagnosis, since survival rates and the

**Table XI** Studies of childhood cancer

| *Reference* | *Years of diagnosis* | *Setting* | *No. of patients* | *Diagnostic group* | *Variables studied* | *Outcome measures* |
|---|---|---|---|---|---|---|
| MRC (1971) | 1963–67 | England and Wales | 879 | ALL | Specialist centres; trial protocol | Median survival |
| Meadows *et al.* (1983) | 1970–75 | Greater Delaware Valley, USA | 327 | ALL | Specialist centres national protocol | Actuarial survival |
| Stiller & Draper (1989) | 1971–84 | Great Britain | 4,697 | ALL | Patients per hospital; entry to trials | Actuarial survival |
| Stiller & Eatock (1994) | 1975–88 | Great Britain | 818 | ANLL | Type of hospital; entry to trials | Actuarial survival |
| Lennox *et al.* (1975) | 1962–68 | Great Britain | 268 | Retinoblastoma | Specialist centres | Four year survival |
| Sanders *et al.* (1988) | 1969–80 | Great Britain | 431 | Retinoblastoma | Specialist centres | Three year survival |
| Lennox *et al.* (1979) | 1970–73 | Great Britain | 313 | Wilms' tumour | Specialist centres; entry to trial | Three year survival |
| Griffel (1977) | 1950–72 | New York, USA | 127 | Wilms' tumour | Specialist centres | Five year survival |
| Duffner *et al.* (1982) | 1968–79 | Connecticut, USA | 278 | Brain tumours | Specialist centres | Actuarial survival |
| Stiller & Lennox (1983) | 1971–77 | Great Britain | 368 | Medulloblastoma | Patients per hospital | Actuarial survival |
| Kramer *et al.* (1984) | 1970–79 | Greater Delaware Valley, USA | 147<br>76<br>87 | Wilms' tumour<br>Medulloblastoma<br>Rhabdomyosarcoma | Specialist centres | Actuarial survival |
| Stiller (1988) | 1977–84 | Great Britain | 435<br>497<br>486<br>483<br>240<br>234<br>351 | Hodgkin's disease<br>NHL<br>Neuroblastoma<br>Wilms' tumour<br>Osteosarcoma<br>Ewing's sarcoma<br>Rhabdomyosarcoma | Specialist centres | Actuarial survival |
| Mann & Stiller (1994) | 1977–91 | Great Britain | 555 | Germ cell | Specialist centres | Actuarial survival |

proportion of children referred to paediatric oncology centres both varied during the study period. The difference was highly significant for boys with testicular tumours, for whom the 5 year survival rate at paediatric oncology centres during 1987–91 was 100%.

In New York State during 1960–72 children with Wilms' tumour who lived in and around Buffalo were more likely to be treated at hospitals with larger numbers of Wilms' tumour patients and also had a substantially higher survival rate (Griffel, 1977). In Connecticut during 1968–77, the survival rate was higher at university cancer centres for children with medulloblastoma or brain stem glioma but not for ependymoma or astrocytoma (Duffner *et al.*, 1982). In the Greater Delaware Valley during 1970–79, children with medulloblastoma or rhabdomyosarcoma had a significantly higher 3 year disease-free survival rate if they were treated at a specialist cancer centre, but survival did not vary between types of hospital for children with Wilms' tumour (Kramer *et al.*, 1984).

### Discussion

Although there is at first sight an impressively large number of studies of survival of cancer patients in relation to patterns of organisation of medical care, the results of different studies can hardly ever be formally pooled to produce a new, more precise estimate of the effect of the factors being investigated. Very few studies have been made of these questions in relation to any one type of cancer – in the present review, the largest number found for any site was seven. Two studies of the same site seldom ask the same questions. Studies have taken place over a period of decades and in a wide range of settings. While the effectiveness of particular treatments may be relatively constant with respect to time or geographical location, it is less safe to assume that the same will be true for the effects of patterns of organisation of care.

### *Sources of bias*

Studies of survival rates can be compromised by several types of bias. If patients with a better prognosis are selectively referred to major centres, as was found in a study of colorectal cancer at Columbia University Comprehensive Cancer Center (Neugut *et al.*, 1991), this could produce an artificial appearance of a better chance of survival at those centres. Similarly, patients included in trials may have a higher survival rate than those who are not because many of the excluded patients are ineligible, often because of poor prognostic features, as was found for example in the study of stomach cancer by Ward *et al.* (1992). For these reasons, more weight should be attached to studies in which some attempt was made to adjust for prognostic factors which might be confounders in any analysis of survival in relation to patterns of care. Even then, results need to be interpreted with caution. If patients at large centres undergo more thorough investigation, some may be assigned to a higher stage than if they had been treated at another hospital, as was found in the study of lung cancer by Greenberg *et al.* (1991). Therefore, measures of disease severity which are likely to be the same for an individual patient regardless of where that patient is treated are preferable; these include white cell count for leukaemia and performance status (Karnofsky & Burchenal, 1949) for a wide range of cancers. Limiting the analysis to patients who survive for at least a specified short period after diagnosis, or long enough to receive a particular type of treatment, can also reduce bias by excluding many subjects who probably had little chance of remission. This approach, however, is obviously inappropriate for studying short-term mortality following surgery.

Some of the problems of referral bias which arise when patients are compared on the basis of hospital of treatment can be avoided by using instead a classification based on area of residence, as in the series of studies from Finland. Interpretation is still problematic, however, as areas containing specialist treatment centres may be of a different socioeconomic status. Delay in diagnosis, attributable at least in part to delayed presentation, may vary between areas (Launoy *et al.*, 1992), and the presence of screening programmes can also affect the distribution of disease severity between areas (Pickering *et al.*, 1992).

There is also the possibility of publication bias, whereby studies whose conclusions point in a particular direction are more likely to appear in journals, but for this review this has been dealt with to some extent by obtaining statements from cancer registries whose data had been used in published studies that they had not also contributed to other studies which had remained unpublished.

### *Effects of hospital and protocol*

Despite the limitations discussed above, there are some discernible patterns in the material reviewed here. A substantial proportion of studies reported higher survival rates for patients who were treated at major centres dealing with larger numbers of cases or at teaching hospitals and other specialist centres or for patients who were treated according to standard protocols, usually within trials. The most obvious explanation for these results is that greater clinical experience and standardisation of treatment are likely to produce higher survival rates. There was no consistent pattern among the studies reviewed of this effect being limited to rare or common cancers, or to cancers with an especially good or poor prognosis.

Although patients treated at specialist centres or in trials frequently have higher survival rates, and there is no evidence that such treatment results in a lower survival rate, there might nevertheless be concern that improvements in survival which are often associated with more intensive therapy are counterbalanced by an increase in treatment-related morbidity. Causes of death have hardly ever been studied in relation to patterns of care. Among children with non-Hodgkin's lymphoma treated in Britain during 1977–85, treatment-related mortality, at least in the medium term, was no higher at paediatric oncology centres than elsewhere (Robertson *et al.*, 1992).

For some tumours, particularly those with a good prognosis, non-protocol treatment can be more intensive. Among residents of the West Midlands region of England treated for parotid pleomorphic adenoma during 1977–86, the proportion of patients receiving radiotherapy, which is not now recommended for this disease, ranged from zero to 57% among the nine centres treating at least one patient a year during the study period (Parry *et al.*, 1993). During 1980–82, children with Wilms' tumour in Britain who were neither included in the national trial nor treated by a paediatric oncologist tended to be overtreated by comparison with current recommendations, mostly by receiving more radiotherapy than would have been given had they been included in the trial (Pritchard *et al.*, 1989). In both of these examples, the apparently unnecessary radiotherapy could give rise to second malignancies and other late effects.

Few studies have investigated the effect of size or type of treatment centre on the survival of patients in clinical trials or treated on protocols. In the United States, no difference was found in survival rates between major centres and local community hospitals for any of the nine cancer sites included in trials sponsored by the Eastern Cooperative Oncology Group during 1976–81 (Begg *et al.*, 1982), or for five studies of the Radiation Therapy Oncology Group (Gillespie *et al.*, 1986). In both the British and American studies of childhood ALL there was no indication that survival rates for children entered in trials or treated according to standard protocols varied with size or type of treatment centre (Meadows *et al.*, 1983; Stiller & Draper, 1989); an unusually high survival rate was achieved at one centre in Britain during the 1970s which had only a moderate number of patients, but where there was believed to be an unusually strong emphasis for that period on strict adherence to the treatment protocol (Eden *et al.*,

1988). Two studies of testicular cancer found that, among men receiving protocol treatment, survival rates were higher at a single centre treating very large numbers of patients (Aass *et al.*, 1991; Harding *et al.*, 1993), but this could equally be due to size of centre or to some other attribute.

*Studies of individual clinicians*

In addition to the studies of survival in relation to size or type of treatment centre reviewed here, there have been a number of studies in which patients have been categorised according to measures of experience of the treating surgeon. Studies of oesophageal and gastric cancer in the West Midlands region of England have indicated that short-term survival is better for the patients of surgeons who do larger numbers of operations (Matthews *et al.*, 1986; Allum *et al.*, 1989). Two studies of colorectal cancer have shown wide variability in mortality between surgeons (Phillips *et al.*, 1984; McArdle & Hole, 1991), but this appeared to be unrelated to the number of operations performed. Studies of survival in relation to seniority of surgeon may be especially problematic since if they were to show a higher survival rate for patients operated on by consultants then this might suggest that such operations should wherever possible be carried out by consultants, but such a policy would be irreconcilable with the fact that the junior surgeons undergoing training today are the consultants of tomorrow. In studies of colorectal and bladder cancer, however, mortality was similar among patients operated on by consultants and trainee surgeons after allowance was made for disease severity (Phillips *et al.*, 1984; Gulliford *et al.*, 1991).

*Rare and common cancers*

In the absence of formal comparative studies it has been suspected for some time, on the basis of uncontrolled comparison of national survival rates or of those from non-specialist units, that treatment of rare cancers such as testicular tumours in specialist units results in higher survival rates (Bagshawe *et al.*, 1985; Thornhill *et al.*, 1988*b*). It is now widely accepted that rare cancers, particularly those for which there have been substantial improvements in the effectiveness of treatment, should be treated in specialist centres. It is sometimes felt, however, that treatment of common cancers need not be organised in the same way (Kingston *et al.*, 1992). In the present review there are several examples of common cancers for which treatment in a specialist centre, usually a teaching hospital, apparently conferred no benefit. This is perhaps not surprising if the higher survival rate for rare cancers at specialist centres is really due to greater clinical experience, since many district hospitals will also have treated large numbers of patients with cancers of sites such as the bowel, lung and breast. Studies relating survival to the number of patients seen at a hospital rather than to the type of hospital may well sometimes be more relevant. For ovarian carcinoma, however, a relatively common cancer but one for which there have been important developments in therapy, the studies reviewed above indicate that specialist treatment is associated with higher survival.

*Socioeconomic and demographic factors*

Changes in the provision of health care, particularly with the containment of costs as a primary objective, can give rise to anxiety over the effects on equity, i.e. the principle that outcome should as far as possible be unaffected by such factors as area of residence, social class, ethnic group or age (Pollock, 1993). Survival rates may vary between areas as a result of delayed presentation which could be due to deficiencies in the effectiveness of screening, primary care or health education. They could also, however, vary because of differences in treatment if patients are more likely to be referred to a major centre if they live closer to that centre or are of higher socioeconomic status. Many studies have found variations in survival with social class; the relative contributions of patient-related and treatment-related factors are often unknown, but it seems likely that delayed presentation among people of low socioeconomic status accounts for much of the difference (Kogevinas *et al.*, 1991). In the United States, blacks have a poorer survival rate than whites for many cancer sites (Howard *et al.*, 1992). Part of the difference is accounted for by lower socioeconomic status and later presentation, but again the contribution of variations in patterns of care to differences in survival between ethnic groups is unknown.

The treatment of cancer in the elderly has provoked controversy (Fentiman, 1991). Nearly all of the studies reviewed here had an upper age limit, and there is no evidence as to the effect of patterns of organisation of care on survival rates specifically for the elderly. Most trials have themselves excluded older people, often because it was felt that they would not withstand intensive treatment, with the result that there is little objective evidence as to what is the best treatment for them. Among women enrolled in trials of breast cancer in the south-eastern United States, response rates, survival and toxicity were all similar for patients aged under or over 70 (Christman *et al.*, 1992). Lack of access to transport can often be a reason for undertreatment of elderly patients (Goodwin *et al.*, 1993).

*Other outcome measures*

This review has concentrated on studies of survival rates that have death or, less frequently, recurrence as the end point. Survival is often both the most important and the most easily measurable outcome. For many cancers, however, particularly those of advanced stage and extremely poor prognosis, treatment is essentially palliative and substantial variations in survival rates are unlikely to occur. There are apparently no published studies of palliation and quality of life in relation to patterns of organisation of care for these patients, and this is clearly an important area for future research.

For cancers with a relatively good prognosis, quality of life as well as absolute survival is also important, though again there appears to be little published research in relation to organisational factors. A population-based study in Washington State, USA, found that women with early-stage breast cancer were less likely to receive breast-conserving surgery and radiotherapy rather than mastectomy if they lived outside the region's major urban centre and particularly if they lived in a county without its own radiotherapy facilities (Lazovich *et al.*, 1991). In a study of women with early-stage breast cancer treated at 19 British hospitals, however, there were no significant differences in the incidence of anxiety and depression between women who had breast-conserving surgery or mastectomy (Fallowfield *et al.*, 1990). The rates of psychiatric morbidity in both treatment groups were similar to those observed in a previous randomised trial of the two forms of treatment.

### Conclusions

In conclusion, referral to a specialist centre or to a hospital treating many patients with the disease, or inclusion in a clinical trial, is often linked with a higher survival rate for the cancers which have been studied; there is no evidence that centralised referral or treatment according to protocols leads to lower survival rates. Some published studies antedate the introduction of current methods of treatment, and research has also been carried out in populations covered by a wide range of health care systems. In order to establish the cancer types for which standardised referral and treatment are most beneficial, and to monitor the effects on survival of new arrangements for the organisation of medical care, further research is needed. Population-based cancer registries, covering an increasing proportion of the world's population, are an invaluable source of data for such studies. In Britain, further studies will be facilitated if the cancer registration system adopts the recommendation of the most recent national review committee that lists of patients enrolled in trials are linked with regional and national registries (Working Group of the Registrar General's Advisory Committee, 1990).

I thank the many directors and other staff in cancer registries who brought to my attention studies which might otherwise have been missed. I am grateful to Mrs E.M. Roberts for secretarial assistance and to Mrs M.B. Allen for her work on the library containing all the publications reviewed here. The Childhood Cancer Research Group is supported by the Department of Health and the Scottish Home and Health Department.

**References**

AASS, N., KLEPP, O., CAVALLIN-STAHL, E., DAHL, O., WICKLUND, H., UNSGAARD, B., BALDETORP, L., AHLSTRÖM, S. & FOSSA, S.D. (1991). Prognostic factors in unselected patients with nonseminomatous metastatic testicular cancer: a multicenter experience. *J. Clin. Oncol.*, **9**, 818-826.

ALLUM, W.H., POWELL, D.J., McCONKEY, C.C. & FIELDING, J.W.L. (1989). Gastric cancer: a 25-year review. *Br. J. Surg.*, **76**, 535-540.

BAGSHAWE, K.D., BEGENT, R.H.J., NEWLANDS, E.S. & RUSTIN, G.J.S. (1985). What sort of oncology team should treat testicular teratoma? *Lancet*, **i**, 930.

BASNETT, I., GILL, M. & TOBIAS, J.S. (1992). Variations in breast cancer management between a teaching and a non-teaching district. *Eur. J. Cancer*, **28A**, 1945-1950.

BEGG, C.B., CARBONE, P.P., ELSON, P.J. & ZELEN, M. (1982). Participation of community hospitals in clinical trials. Analysis of five years of experience in the Eastern Cooperative Oncology Group. *N. Engl. J. Med.*, **306**, 1076-1080.

BERTELSEN, K. (1991). Protocol allocation and exclusion in two Danish randomised trials in ovarian cancer. *Br. J. Cancer*, **64**, 1172-1176.

BOFFETTA, P., MERLETTI, F., WINKELMANN, R., MAGNANI, C., CAPPA, A.P.M. & TERRACINI, B. (1993). Survival of breast cancer patients from Piedmont, Italy. *Cancer Causes Control*, **4**, 209-215.

BONETT, A., RODER, D. & ESTERMAN, A. (1991). Case-survival rates for infiltrating ductal carcinomas by category of hospital at diagnosis in South Australia. *Med. J. Aust.*, **154**, 695-697.

BREWER, J.I., ECKMAN, T.R., DOLKART, R.E., TOROK, E.E. & WEBSTER, A. (1971). Gestational trophoblastic disease. A comparative study of the results of therapy in patients with invasive mole and with choriocarcinoma. *Am. J. Obstet. Gynecol.*, **109**, 335-340.

CHOUILLET, A.M., BELL, C.M.J. & HISCOX, J.G. (1994). Management of breast cancer in southeast England. *Br. Med. J.*, **308**, 168-171.

CHRISTMAN, K., MUSS, H.B., CASE, L.D. & STANLEY, V. (1992). Chemotherapy of metastatic breast cancer in the elderly. The Piedmont Oncology Association experience. *JAMA*, **268**, 57-62.

CHU, J., POLISSAR, L. & TAMIMI, H.K. (1982). Quality of care in women with stage I cervical cancer. *West. J. Med.*, **137**, 13-17.

DAVIS, S., WRIGHT, P.W., SCHULMAN, S.F., HILL, L.D., PINKHAM, R.D., JOHNSON, L.P., JONES, T.W., KELLOGG, H.B., RADKE, H.M., SIKKEMA, W.W., JOLLY, P.C. & HAMMAR, S.P. (1985). Participants in prospective, randomized clinical trials for resected non-small cell lung cancer have improved survival compared with nonparticipants in such trials. *Cancer*, **56**, 1710-1718.

DAVIS, S., DAHLBERG, S., MYERS, M.H., CHEN, A. & STEINHORN, S.C. (1987). Hodgkin's disease in the United States: a comparison of patient characteristics and survival in the Centralized Cancer Patient Data System and the Surveillance, Epidemiology, and End Results Program. *J. Natl Cancer Inst*, **78**, 471-478.

DIAMOND, J.J., STEINFELD, A.D. & HANKS, G.E. (1991). The relationship between facility structure and outcome in cancer of the prostate and uterine cervix. *Int. J. Radiat. Oncol. Biol. Phys.*, **21**, 1085-1087.

DUFFNER, P.K., COHEN, M.E. & FLANNERY, J.T. (1982). Referral patterns of childhood brain tumors in the State of Connecticut. *Cancer*, **50**, 1636-1640.

EBELING, K., TANNEBERGER, S.T., NISCHAN, P., JAROFKE, D. & KLUGE, E. (1982). Bösartige Neubildungen der weiblichen Brustdrüse in der Hauptstadt der DDR, Berlin, in der Periode 1975 bis 1979. *Arch. Geschwulstforsch.*, **52**, 307-321.

EDEN, O.B., STILLER, C.A. & GERRARD, M.P. (1988). Improved survival for childhood acute lymphoblastic leukemia: possible effect of protocol compliance. *Pediatr. Hematol. Oncol.*, **5**, 83-91.

EDGE, S.B., SCHMIEG, R.E., ROSENLOF, L.K. & WILHELM, M.C. (1993). Pancreas cancer resection outcome in American university centers in 1989-1990. *Cancer*, **71**, 3502-3508.

EISENKOP, S.M., SPIRTOS, N.M., MONTAG, T.W., NALICK, R.H. & WANG, H.-J. (1992). The impact of subspecialty training on the management of advanced ovarian cancer. *Gynecol. Oncol.*, **47**, 203-209.

FALLOWFIELD, L.J., HALL, A., MAGUIRE, G.P. & BAUM, M. (1990). Psychological outcomes of different treatment policies in women with early breast cancer outside a clinical trial. *Br. Med. J..*, **301**, 575-580.

FENTIMAN, I.S. (1991). Treatment of cancer in the elderly. *Br. J. Cancer*, **64**, 993-995.

GILL, M., McCARTHY, M., MURRELLS, T. & SILCOCKS, P. (1988). Chemotherapy for the primary treatment of osteosarcoma: population effectiveness over 20 years. *Lancet*, **i**, 689-692.

GILLESPIE, B.W., DIAMOND, J.J., DAVIS, L.W. & ROMINGER, C.J. (1986). An outcome study of the RTOG cancer control program. *Int. J. Radiat. Oncol. Biol. Phys.*, **12**, 2157-2163.

GILLIS, C.R., HOLE, D.J., STILL, R.M., DAVIS, J. & KAYE, S.B. (1991). Medical audit, cancer registration, and survival in ovarian cancer. *Lancet*, **337**, 611-612.

GOODWIN, J.S., HUNT, W.C. & SAMET, J.M. (1993). Determinants of cancer therapy in elderly patients. *Cancer*, **72**, 594-601.

GREENBERG, E.R., BARON, J.A., DAIN, B.J., FREEMAN, D.H., YATES, J.W. & KORSON, R. (1991). Cancer staging may have different meanings in academic and community hospitals. *J. Clin. Epidemiol.*, **44**, 505-512.

GRIFFEL, M. (1977). Wilms' tumor in New York state: epidemiology and survivorship. *Cancer*, **40**, 3140-3145.

GULLIFORD, M.C., PETRUCKEVITCH, A. & BURNEY, P.G.J. (1991). Survival with bladder cancer, evaluation of delay in treatment, type of surgeon, and modality of treatment. *Br. Med. J.*, **303**, 437-440.

HAKAMA, M., KARJALAINEN, S. & HAKULINEN, T. (1989). Outcome-based equity in the treatment of colon cancer patients in Finland. *Int. J. Technol. Assessment Hlth. Care*, **5**, 619-630.

HANKS, G.E., HERRING, D.F. & KRAMER, S. (1983). Patterns of care outcome studies. Results of the national practice in cancer of the cervix. *Cancer*, **51**, 959-967.

HANKS, G.E., DIAMOND, J.J. & KRAMER, S. (1985). The need for complex technology in radiation oncology. Correlations of facility characteristics and structure with outcome. *Cancer*, **55**, 2198-2201.

HARDING, M.J., PAUL, J., GILLIS, C.R. & KAYE, S.B. (1993). Management of malignant teratoma: does referral to a specialist unit matter? *Lancet*, **341**, 999-1002.

HARLAN, L.C. (1992). Special data collection for treatment patterns. In *Cancer Statistics Review 1973-1989*, Miller, B.A., Ries, L.A.G., Hankey, B.F., Kosary, C.L. & Edwards, B.K. (eds) pp. XXXI 1-5, NIH Publication No. 92-2789. National Cancer Institute: Bethesda.

HOGBERG, T., CARSTENSEN, J. & SIMONSEN, E. (1993). Treatment results and prognostic factors in a population-based study of epithelial ovarian cancer. *Gynecol. Oncol.*, **48**, 38-49.

HOWARD, J., HANKEY, B.F., GREENBERG, R.S., AUSTIN, D.F., CORREA, P., CHEN, V.W. & DURAKO, S. (1992). A collaborative study of differences in the survival rates of black patients and white patients with cancer. *Cancer*, **69**, 2349-2360.

JUNOR, E.J., HOLE, D.J. & GILLIS, C.R. (1994). Management of ovarian cancer: referral to a multidisciplinary team matters. *Br. J. Cancer*, **70**, 363-370.

KARJALAINEN, S. (1990). Geographical variation in cancer patient survival in Finland: chance, confounding, or effect of treatment? *J. Epidemiol. Community Hlth.*, **44**, 210-214.

KARJALAINEN, S. & PALVA, I. (1989). Do treatment protocols improve end results? A study of survival of patients with multiple myeloma in Finland. *Br. Med. J.*, **299**, 1069-1072.

KARJALAINEN, S. & PUKKALA, E. (1990). Social class as a prognostic factor in breast cancer survival. *Cancer*, **66**, 819-826.

KARNOFSKY, D.A. & BURCHENAL, J.H. (1949). The clinical evaluation of chemotherapeutic agents in cancer. In *Evaluation of Chemotherapeutic Agents Symposium*, Macleod, C.M. (ed.) pp. 191-250. Columbia University Press: New York.

KINGSTON, R.D., WALSH, S. & JEACOCK, J. (1992). Colorectal surgeons in district general hospitals produce similar survival outcomes to their teaching hospital colleagues: review of 5-year survivals in Manchester. *J. R. Coll. Surg. Edin.*, **37**, 235-237.

KOGEVINAS, M., MARMOT, M.G., FOX, A.J. & GOLDBLATT, P.O. (1991). Socioeconomic differences in cancer survival. *J. Epidemiol. Community Hlth.*, **45**, 216-219.

KRAMER, S. (1981). An overview of process and outcome data in the patterns of care study. *Int. J. Radiat. Oncol. Biol. Phys.*, **7**, 795-800.

KARMER, S., MEADOWS, A.T., PASTORE, G., JARRETT, P. & BRUCE, D. (1984). Influence of place of treatment on diagnosis, treatment, and survival in three pediatric solid tumors. *J. Clin. Oncol.*, **2**, 917-923.

LANCIANO, R.M., WON, M., COIA, L.R. & HANKS, G.E. (1991). Pretreatment and treatment factors associated with improved outcome in squamous cell carcinoma of the uterine cervix: a final report of the 1973 and 1978 patterns of care studies. *Int. J. Radiat. Oncol. Biol. Phys.*, **20**, 667-676.

LAUNOY, G., LE COUTOUR, X., GIGNOUX, M., POTTIER, D. & DUGLEUX, G. (1992). Influence of rural environment on diagnosis, treatment, and prognosis of colorectal cancer. *J. Epidemiol. Community Hlth.*, **46**, 365-367.

LAZOVICH, E., WHITE, E., THOMAS, D.B. & MOE, R.E. (1991). Underutilization of breast-conserving surgery and radiation therapy among women with stage I or II breast cancer. *JAMA*, **266**, 3433-3438.

LEIBEL, S.A., HANKS, G.E. & KRAMER, S. (1984). Patterns of care outcome studies: results of the national practice in adenocarcinoma of the prostate. *Int. J. Radiat. Oncol. Biol. Phys.*, **10**, 401-409.

LENNOX, E.L., DRAPER, G.J. & SANDERS, B.M. (1975). Retinoblastoma: a study of natural history and prognosis of 268 cases. *Br. Med. J.*, **3**, 731-734.

LENNOX, E.L., STILLER, C.A., MORRIS JONES, P.H. & KINNIER WILSON, L.M. (1979). Nephroblastoma: treatment during 1970-3 and the effect on survival of inclusion in the first MRC trial. *Br. Med. J.*, **ii**, 567-569.

LIBERATI, A., CONFALONIERI, C., ANDREANI, A., COLOMBO, F., FRANCESCHI, S., LA VECCHIA, C., TALAMINI, R. & TOGNONI, G. (1983). Lung cancer care in general hospitals. *Tumori*, **69**, 567-573.

LIBERATI, A., MANGIONI, C., BRATINA, L., CARINELLI, G., MARSONI, S., PARAZZINI, F., REGALLO, M., TALAMINI, R. & TOGNONI, G. (1985). Process and outcome of care for patients with ovarian cancer. *Br. Med. J.*, **291**, 1007-1012.

LUSTIG, R.A., MACLEAN, C.J., HANKS, G.E. & KRAMER, S. (1984). The patterns of care outcome studies: results of the national practice in carcinoma of the larynx. *Int. J. Radiat. Oncol. Biol. Phys.*, **10**, 2357-2362.

LUSTIG, R.A., KRALL, J.M., CURRAN, W.J. & HANKS, G.E. (1991). Improvements observed in care and outcome in carcinoma of the larynx. *Int. J. Radiat. Oncol. Biol. Phys.*, **20**, 101-104.

MCARDLE, C.S. & HOLE, D. (1991). Impact of variability among surgeons on postoperative morbidity and mortality and ultimate survival. *Br. Med. J.*, **302**, 1501-1505.

MANN, J.R. & STILLER, C.A. (1994). Changing pattern of incidence and survival in children with germ cell tumours. *Advance Biosci.*, **91**, 59-64.

MARSHALL, J.R. & FUNCH, D.P. (1983). Social environment and breast cancer: a cohort analysis of patients' survival. *Cancer*, **52**, 1546-1550.

MATTHEWS, H.R., POWELL, D.J. & MCCONKEY, C.C. (1986). Effect of surgical experience on the results of resection for oesophageal carcinoma. *Br. J. Surg.*, **73**, 621-623.

MEADOWS, A.T., KRAMER, S., HOPSON, R., LUSTBADER, E., JARRETT, P. & EVANS, A.E. (1983). Survival in childhood acute lymphocytic leukemia: effect of protocol and place of treatment. *Cancer Invest.*, **1**, 49-55.

MÖHNER, M. & SLISOW, W. (1990). Untersuchung zum Einfluss der regional zentralisierten Behandlung auf die Überlebenschancen beim Rektumkarzinom in der DDR. *Zentralbl. Chir.*, **115**, 801-812.

MRC (MEDICAL RESEARCH COUNCIL) (1971). Duration of survival of children with acute leukaemia. *Br. Med. J.*, **4**, 7-9.

NEUGUT, A.I., TIMONY, D. & MURRAY, T. (1991). Colorectal cancer: differences between community and geographically distant patients seen at an urban medical center. *Dis. Colon Rectum*, **34**, 64-68.

NGUYEN, H.N., AVERETTE, H.E., HOSKINS, W., PENALVER, M., SEVIN, B.-U. & STEREN, A. (1993). National Survey of Ovarian Carcinoma Part V. The impact of physician's specialty on patients' survival. *Cancer*, **72**, 3663-3670.

PARRY, J., WILSON, S., CUMMINS, C., REDMAN, V. & WOODMAN, C. (1993). A review of parotid pleomorphic adenomas in the West Midlands Region 1977-1986. *Clin. Oncol.*, **5**, 147-149.

PHILLIPS, R.K.S., HITTINGER, R., BLESOVSKY, L., FRY, J.S. & FIELDING, L.P. (1984). Local recurrence following 'curative' surgery for large bowel cancer. 1. The overall picture. *Br. J. Surg.*, **71**, 12-16.

PICKERING, R.M., CHADWELL, I.R. & MOUNTNEY, L. (1992). Importance of district of residence and known primary site for bowel cancer survival: analysis of data from Wessex Cancer Registry. *J. Epidemiol. Community Hlth.*, **46**, 266-270.

POLLOCK, A.M. (1993). The future of health care in the United Kingdom. *Br. Med. J.*, **306**, 1703-1704.

PRITCHARD, J., STILLER, C.A. & LENNOX, E.L. (1989). Overtreatment of children with Wilms' tumour outside paediatric oncology centres. *Br. Med. J..*, **299**, 835-836.

ROBERTSON, C.M., STILLER, C.A. & KINGSTON, J.E. (1992). Causes of death in children diagnosed with non-Hodgkin's lymphoma between 1974 and 1985. *Arch. Dis. Child.*, **67**, 1378-1383.

ROMANO, P.S. & MARK, D.H. (1992). Patient and hospital characteristics related to in-hospital mortality after lung cancer resection. *Chest*, **101**, 1332-1337.

SANDERS, B.M., DRAPER, G.J. & KINGSTON, J.E. (1988). Retinoblastoma in Great Britain 1969-80: incidence, treatment, and survival. *Br. J. Ophthalmol.*, **72**, 576-583.

SILAGY, C. (1993). Developing a register of randomised controlled trials in primary care. *Br. Med. J.*, **306**, 897-900.

SLISOW, W., MARX, G., SEIFART, W. & STANECZEK, W. (1987). Argumentation für eine regionale zentralisierte Behandlung beim Magenkarzinom Eine statistiche Untersuchung von nationalen Resultaten. *Zentralbl. Chir.*, **112**, 27-33.

STILLER, C.A. (1988). Centralisation of treatment and survival rates for cancer. *Arch. Dis. Child.*, **63**, 23-30.

STILLER, C.A. (1992). Survival of patients in clinical trials and at specialist centres. In *New Treatments for Cancer: Practical, Ethical and Legal Problems*, Williams, C.J. (ed.) pp. 119-136. Wiley: Chichester.

STILLER, C.A. & DRAPER, G.J. (1989). Treatment centre size, entry to trials, and survival in acute lymphoblastic leukaemia. *Arch. Dis. Child.*, **64**, 657-661.

STILLER, C.A. & EATOCK, E.M. (1994). Survival from acute non-lymphocytic leukaemia 1971-88: a population-based study. *Arch. Dis. Child.*, **70**, 219-223.

STILLER, C.A. & LENNOX, E.L. (1983). Childhood medulloblastoma in Britain 1971-77: analysis of treatment and survival. *Br. J. Cancer*, **48**, 835-841.

THORNHILL, J.A., WALSH, A., CONROY, R.M., FENNELLY, J.J., KELLY, D.G. & FITZPATRICK, J.M. (1988*a*). Physician-dependent prognostic variables in the management of testicular cancer. *Br. J. Urol.*, **61**, 244-249.

THORNHILL, J.A., WALSH, A., KELLY, D., FENNELLY, J.J. & FITZPATRICK, J.M. (1988*b*). Non-seminomatous germ cell testis cancer in Ireland (1980-1985). Management, results and prognostic variables with reference to national management protocols. *Eur. Urol.*, **15**, 84-88.

WARD, L.C., FIELDING, J.W.L., DUNN, J.A. & KELLY, K.A. (1992). The selection of cases for randomised trials: a registry survey of concurrent trial and non-trial patients. *Br. J. Cancer*, **66**, 943-950.

WORKING GROUP OF THE REGISTRAR GENERAL'S MEDICAL ADVISORY COMMITTEE (1990). *Review of the National Cancer Registration System*, Office of Population Censuses and Surveys. Series MB1, No. 17. HMSO: London.